# Assessing the Overlap Between Three Measures of Food Reward

**DOI:** 10.3389/fpsyg.2019.00883

**Published:** 2019-04-26

**Authors:** Kadri Arumäe, Kairi Kreegipuu, Uku Vainik

**Affiliations:** ^1^Institute of Psychology, University of Tartu, Tartu, Estonia; ^2^Montreal Neurological Institute and Hospital, McGill University, Montreal, QC, Canada

**Keywords:** food reward, Leeds Food Preference Questionnaire, grip force, emotional attentional blink, hunger, binge eating, snack food calorie intake

## Abstract

Food reward is an important concept for research in eating behaviors. Many food reward tasks have been developed and are in active use. However, little is known how much these tasks overlap. Here, we sought to compare three promising food reward tasks: (1) the Leeds Food Preference Questionnaire (LFPQ; a procedure combining explicit ratings of wanting and liking and an implicit wanting task based on forced choice), (2) a hand grip force task, and (3) an emotional attentional blink (EAB) task. Specifically, we assessed whether the tasks are sensitive to changes in hunger, correlate with each other, and correlate with trait binge eating and snack food calorie intake. Thirty-nine women aged 25.51 ± 5.99 years, with a BMI of 22.51 ± 3.58 kg/m^2^ completed the three tasks twice: after a 6-h fast and following a breakfast meal. In the fasted condition, participants were also given *ad libitum* access to snack foods to assess calorie intake. Prior to the two laboratory sessions, participants completed a trait binge eating questionnaire. Results revealed that the LFPQ’s explicit wanting and explicit liking subscales, as well as grip force reflected higher food reward scores in the fasted condition. The three metrics also correlated positively with each other. Explicit wanting and liking correlated with snack food intake, while grip force did not. None of the tasks were related to trait binge eating. Reaction times in the forced choice procedure did not reflect changes in hunger, but the task was nevertheless able to differentiate between foods varying in taste and fat content. The EAB was not sensitive to the hunger manipulation; neither did the task correlate with binge eating or energy intake. Collectively, our findings suggest that the explicit wanting and liking scales and the grip force task measure the same construct, whereas EAB results may be obscured by a variety of potential confounding factors. Future research could include additional food reward tasks in comparisons, measure covariates that may moderate the variables’ associations, and compare hunger-dependent changes in food reward in different subgroups.

## Introduction

Increased rates for obesity have motivated a sizable literature investigating the psychological aspects of overeating. While a variety of other factors contribute to eating-related decision-making (Vainik et al., 2013; Emery and Levine, 2017; Michaud et al., 2017; Stevenson, 2017; Higgs and Spetter, 2018; Yang et al., 2018), food reward has gained extensive attention from researchers due to its significant potential to predict energy intake (Temple, 2014; Rogers and Hardman, 2015). Food reward describes the value of a specific food to a person at a particular moment. The brain’s reward system is the mechanism that motivates activity to seek out food and other biologically relevant stimuli and gives rise to the experience of pleasure once the reward is obtained or consumed. Reward is processed separately from the sensory properties of a food in the brain: a decrease in hunger causes a drop in the reward value of food, but taste and olfactory processing remain unchanged (Rolls, 2015). Because the reward system is related to the mechanisms that influence food intake beyond homeostatic need (Alonso-Alonso et al., 2015), differences in responsivity to food should explain some of the variability in food intake and predict maladaptive eating (Temple, 2014; Rogers and Hardman, 2015).

The distinguishable processes called wanting and liking have separate roles in food reward, with the former being a state-dependent, motivational component, and the latter a hedonic component associated with the experience of pleasure. A variety of approaches have been employed to measure food reward and its components in humans. A key difference of these tasks is their degree of explicitness. Most explicit procedures rely on subjective report: participants give ratings about their liking or wanting for a food after tasting it (Rogers and Hardman, 2015) or after seeing its image on a computer screen. A popular example of the latter approach is The Leeds Food Preference Questionnaire (LFPQ; Finlayson et al., 2007), a computerized task that uses visual analog scale (VAS) ratings to measure explicit wanting and explicit liking. Such self-report measures are easy to administer, requiring little time and effort from participants. The LFPQ is reactive to changes in hunger and can differentiate the magnitude of reward for foods that differ by taste and fat content (Finlayson et al., 2008).

Another category of tasks relies on the idea that people are willing to exert effort to obtain rewards, whereas the amount of effort is proportional to the value of the reward. In work-for-food tasks based on this assumption, participants either press the keyboard or mouse (Temple, 2014; Rogers and Hardman, 2015) or squeeze a hand-held dynamometer (Ziauddeen et al., 2014) to express the value a food has for them at the moment of response. As grip force measures have been shown to be reactive to sensory-specific satiety (Ziauddeen et al., 2012), the task is likely able to capture changes in a person’s motivational state. Moreover, it has been proposed that the effort exerted through grip force can capture food reward more objectively than self-report (Ziauddeen et al., 2014).

Another class of tools measures food reward on a subliminal or implicit level. These tasks can correct for potential response bias and may capture variability in food reward that explicit ones cannot. For instance, automatic, bottom-up attention can influence eating behavior, but explicit self-report tasks may not be sufficient to capture such processes. However, results using these tasks tend to produce conflicting results, possibly due to the use of different methodologies (Nijs and Franken, 2012; Werthmann et al., 2015; Field et al., 2016). One promising task is built on a robust phenomenon of attention – the emotional attentional blink (EAB) (McHugo et al., 2013), an individual’s reduced ability to notice a target stimulus in a RSVP sequence (Raymond et al., 1992) if it is presented 200–500 ms after an attention-capturing stimulus (Dux and Marois, 2009). A food-specific EAB task has been shown to be responsive to hunger (Piech et al., 2010), demonstrating its ability to capture changes in food-specific motivation. Other tasks relying on reaction times, such as the forced choice procedure of the LFPQ, may also capture an implicit component of food reward.

While food reward tasks are abundant, their convergent validity is mostly unknown. This hampers the generalizability of research conducted with different tasks and can contribute to jangle fallacy – using different names for constructs that reflect the same underlying mechanism (Kelley, 1927). Such jangle fallacy is widespread among eating-related questionnaires that have different names such as Disinhibition, Power of Food, and Binge Eating Scale, but nevertheless can be aggregated into one uncontrolled eating factor (Price et al., 2015; Vainik et al., 2015; Vainik and Meule, 2018). Linking results found with different questionnaires has enabled drawing robust conclusions about the associations that uncontrolled eating has with body mass index, food intake, personality traits, and brain activity (Vainik et al., 2019). Behavioral food reward research could benefit from a similar approach, particularly as behavioral research is more time-consuming than questionnaire-based research. In a recent systematic review on food reward responsiveness to weight loss, studies using different measures were aggregated based on face validity – similarity between explicit food reward measures was assumed (Oustric et al., 2018). However, that assumption would have been stronger if there were covariance data to support the similarity of different measures. Unfortunately, such data is rarely available. There is evidence that a work-for-food paradigm relates to explicit liking, but not to implicit wanting or trait disinhibition (French et al., 2014). We propose that such work needs to be expanded to other prominent food reward tasks.

Here, we sought to test the similarity of three types of food reward tasks: the LFPQ (a questionnaire combining VAS ratings and a forced choice procedure), a hand grip force task, and an EAB task. The tasks were chosen based on their ease of administration and test–retest reliability. The first step was to establish the tasks’ sensitivity to a hunger manipulation. Hunger is a fundamental contributor to wanting and thus the scores of each food motivation task should reflect changes in hunger. In order to establish hunger effects, the three tasks were administered to participants twice: after an overnight fast and following a breakfast meal. To clarify whether hunger affected reactivity to food specifically or had a more general effect on responding, the EAB and grip force tasks included neutral images as a control category. Additionally, each task included stimuli depicting foods that varied in their taste (sweet and savory) and fat content (high-fat and low-fat), allowing for generalization of results across different types of food.

In addition to being sensitive to hunger, if the three tasks truly measure food reward, they should also correlate with each other. The absence of between-task correlations would suggest that they measure different constructs. Moreover, a valid food reward task should correlate with other food reward-related behaviors, such as self-reported uncontrolled eating and energy intake.

Based on these criteria, we hypothesize that when contrasting participants in food-deprived and fed states, participants in a food-deprived state should get higher scores of explicit wanting and liking on the LFPQ, respond slower in the LFPQ’s forced choice procedure, exert more force in the food category of the grip force task, and get fewer correct answers in the food category of the EAB task. We also anticipate that the explicit wanting and liking scores of the LFPQ and grip force in the food category correlate positively with each other and negatively with reaction times in the forced choice task and the percentage of correct answers in the EAB task’s food category. Finally, we expect that trait binge eating and *ad libitum* snack food calorie intake correlate positively with the explicit wanting and liking subscales of the LFPQ and the food category of grip force, and negatively with reaction times in the forced choice procedure and the EAB task’s food category performance.

## Materials and Methods

### Participants

Participants were recruited via social media, a newspaper advertisement, and from among university students in exchange for course credit. Only women were recruited in order to avoid variability arising from gender differences in food cue reactivity (Uher et al., 2006; Wang et al., 2009) and in subjective hunger levels after fasting (Uher et al., 2006). A pre-evaluation questionnaire was used to screen out people with diabetes, those who were taking appetite suppressants, had a diagnosis of an eating disorder or other psychiatric or neurological disorder, were pregnant or breastfeeding, color blind, vegetarian, or had an allergy to an ingredient present in the foods offered during the laboratory sessions. People with corrected vision were asked to wear their glasses or contact lenses during the experiments. A total of forty women participated. All participants confirmed informed consent for participation. The study was approved by the Research Ethics Committee of the University of Tartu.

### Stimuli

A set of images was compiled for the computerized tasks. Images of sweet and savory foods with high and low fat content, found via Internet searches, were rated by 145 people (31 men; mean age = 36.94, *SD* = 15.48 years; mean BMI = 23.16, *SD* = 4.04 kg/m^2^) in an web-based questionnaire. Respondents were asked to give VAS ratings of how much they liked each food and how strong the feeling of liking or disliking was. An optional question allowed respondents to select one image that did not fit in with other images of the same food. Five foods represented each category (high-fat sweet, high-fat savory, low-fat sweet, low-fat savory) and each food was represented by six images. The food items included in the final set are listed in Table 1. The foods with the lowest valence ratings (burger, cookies, crispbread, and gummy candies) were excluded, as well as the individual images that were the least liked and most often marked as different from the others. The different food categories were chosen based on the LFPQ task setup.

**Table 1:** List of foods representing each category in the final set of stimuli.

High-fat savory	High-fat sweet	Low-fat savory	Low-fat sweet
Pizza	Ice cream	Fresh salad	Popsicle
Cheese	Chocolate	Pasta in tomato sauce	Fruit salad
Salted peanuts	Candies	Carrots	Raisins
French fries	Slice of cake	Grilled chicken	Berries


Stimuli for the control categories of the EAB and grip force task were selected from the International Affective Picture System (Lang, 2005). Pictures of household objects (e.g., baskets, cooking pots, oven mitts, scissors, and pens) with neutral valence and low arousal ratings were chosen. Filler pictures for the EAB task depicting cars, boats, airplanes, buses, and bicycles were found via Internet searches.

The final set of stimuli consisted of 64 food, 32 neutral, and 240 filler images. Each picture depicted a food or object in the center on a white background. All images were matched on luminosity.

### Tasks

#### The Leeds Food Preference Questionnaire (LFPQ)

The LFPQ is a computerized task developed to measure explicit wanting and explicit liking via VAS ratings and implicit wanting via a forced choice procedure (Finlayson et al., 2007). The questionnaire has been shown to be sensitive to changes in the physiological state induced by eating (Finlayson et al., 2008) and exercise (Finlayson et al., 2009), suggesting that it is suitable for capturing the changes in food reward that are expected to occur as the individual’s state changes. Both explicit and implicit wanting subscales can predict calorie consumption and food choice (Griffioen-Roose et al., 2011). The test–retest reliability is 0.8–0.9 for the explicit liking subscale and 0.6–0.7 for the implicit wanting subscale (Dalton and Finlayson, 2014). For the current study, the LFPQ was translated into Estonian. Explicit wanting and liking scores are based on ratings of food images on a 100-mm VAS. A total of 16 food images (150 mm × 100 mm) were rated. For explicit wanting, the question “How much do you want this food right now?” was presented above the stimulus. For explicit liking, the question was “How pleasant would it be to taste some of this food now?”. For both scales, the anchor “Not at all” was used on the left side of the scale and “Extremely” on the right side. The trials of the explicit liking and explicit wanting subscale were presented intermittently in a random order. In the implicit wanting procedure, the question “Which food do you most want to eat right now?” was presented, followed by pairs of foods. The participant was instructed to choose the preferred food as fast and accurately as possible by pressing “D” on the keyboard to choose the food on the left, or “J” to choose the food on the right. Each of the 96 pairs was presented for up to 4,000 ms or until a response was given. Between each pair was a break of 500–2000 ms during which a fixation cross was shown in the center of the screen. Reaction times as well as frequency of choice was recorded. The LFPQ was administered on a 17-inch screen using E-Prime 2.0 (Psychology Software Tools, Inc, 2012).

#### Emotional Attentional Blink (EAB)

The emotional attentional blink task (EAB; adapted from Piech et al., 2010) represents a cognitive approach to measuring food reward. In the current study, neutral images were used for comparison to assess whether the effect of the hunger manipulation on attention is general or food-specific. Consecutive image series, each of which consisted of 17 pictures, were presented. Each stimulus appeared for 100 ms. Pictures of transportation devices were used as filler images and pictures of food and household items as distractors. Participants were instructed to pay close attention to the picture series and identify whether the series contained an image of a vehicle turned 90 degrees, and whether the image was rotated clockwise or counterclockwise. Each series contained a distractor. In 75% of the trials, a rotated target stimulus appeared either 2 or 4 images after the distractor. In order to minimize random responding, participants were informed that 25% of trials did not include a rotated image. After each trial, two questions were asked: “Did you see a rotated picture?”, and “Which way was the picture rotated?”. The keys “N” and “C” were used to respond “Yes” or “No” to the first, and the keys “,” and “.” to respond “Clockwise” or “Counterclockwise” to the second question, respectively, on a standard QWERTY keyboard. A total of 192 trials were presented in six blocks, with a chance for a resting break before each new block. Each participant completed a set of 16 practice trials prior to the task in her first laboratory session. The EAB task was administered on a 17-inch screen using E-Prime 2.0 (Psychology Software Tools, Inc, 2012). The outcome variable was the percentage of correct answers for trials that included a rotated picture. Trials with both answers correct were counted as correct. Trials where reaction times for at least one question was too long (>1,000 ms) or too short (<100 ms) were excluded. Although the test–retest reliability of EAB tasks is unknown, domain-general attentional blink paradigms have good test–retest reliability (Kelly and Dux, 2011; Dale and Arnell, 2013).

#### Grip Force Task

The grip force task used is a work-for-food paradigm that operationalizes wanting as the amount of effort an individual is willing to expend to acquire a specific food item. In this task, a handheld dynamometer is squeezed in response to food images to show the degree of motivation, or wanting, for whatever the image depicts. The task was adapted from a study by Ziauddeen et al. (2014). Participants were instructed to exert an amount of effort proportional to the strength of their motivation to eat the depicted food or acquire the pictured non-food object at the moment of response. Pictures of food and household objects were placed on a checkered background of pixel-sized red, brown, green, and yellow squares. Each stimulus was presented for 200 ms in each trial. A patterned mask was presented for a duration of 200 ms before and 100 ms following the stimulus. After stimulus presentation, the words “Squeeze the dynamometer” appeared on the screen to indicate the window of time during which the response was recorded. Participants’ maximum grip force and the baseline measure of the dynamometer were taken prior to the start of the task for calibration purposes. The outcome measure was area under the curve (AUC) that reflects the total amount of force exerted in response to a stimulus. AUC scores were normalized using the following formula: (grip force - baseline)/(maximum grip force - baseline) × 100. A Vernier dynamometer with a measurement range of 0–600 N, an accuracy of ±0.6 N and a resolution of 0.21 N was used. The task was administered using MATLAB, 2006a (MATLAB, 2016) on a 19-inch screen. Domain-general grip strength measures have been shown to have high test–retest reliability (Roberts et al., 2011).

#### Trait Binge Eating

Trait binge eating, an individual’s tendency to exhibit emotions, cognitions, and behaviors characteristic to binge eaters, is a behavioral phenotype of obesity associated with a preference for high-fat sweet food (Dalton and Finlayson, 2014). Additionally, binge eating questionnaires map well to the common construct of uncontrolled eating or reward-related eating (Vainik et al., 2015, 2019; Mason et al., 2017). In the current study, the 9-item binge eating subscale of Eating Disorders Assessment Scale (EDAS; Herik, 2009; Akkermann et al., 2010; also used in Vainik et al., 2015; Sultson et al., 2017) with an internal reliability of 0.86 was used as a measure of trait binge eating. EDAS is a self-report questionnaire designed to assess the symptoms of eating disorders where subjective ratings are given on a six-point Likert scale.

#### Subjective Hunger

Subjective hunger was evaluated with the question “How hungry do you feel right now?”. Answers were recorded on a 100-unit VAS presented on a computer screen with “Not hungry at all” at the left and “Very hungry” at the right side. Subjective hunger was rated on five occasions during each laboratory session.

### Procedure

Prior to the laboratory sessions, participants completed the pre-evaluation questionnaire assessing their suitability for the study, as well as the EDAS. Information about participants’ age, height, and weight was collected via an online form.

Participants individually attended two laboratory sessions beginning at either 8:30 or 10:00 AM, depending on the participant’s preference. The order of the sessions was randomized to eliminate order effects. Both sessions took place in a dimly lit laboratory room. Participants were instructed to refrain from eating or consuming caloric beverages for at least 6 h prior to the beginning of each session. All participants confirmed that they had complied with this instruction.

Both laboratory sessions involved completing the three computerized tasks in a randomized order, as well as giving subjective ratings of hunger on five occasions: upon arrival, and after each phase of the experiment. The fed condition included consuming a test meal before the computerized tasks, consisting of the porridge and the fruit chosen by the participant. Participants were instructed to eat enough of the test meal to make them feel full, but were not required to complete the meal. In the fasted condition, no test meal was offered, but after the completion of the computer tasks they partook in the *ad libitum* snack food calorie intake task where they were instructed to consume as little or as much as they wished.

**FIGURE 1:**
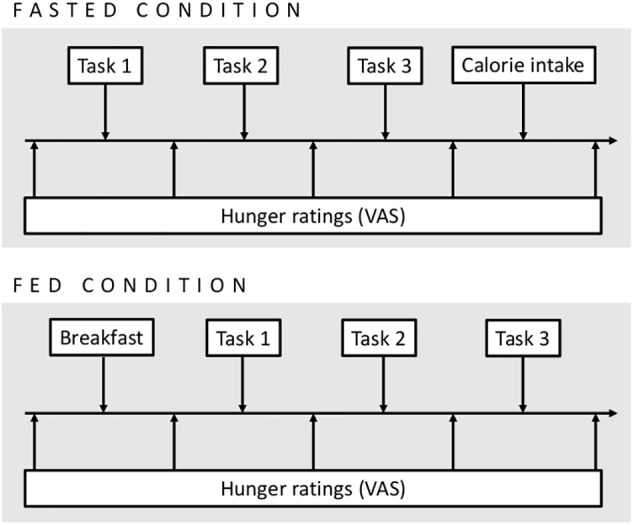
Procedure in the fasted and fed conditions. Tasks 1, 2, and 3 were the Leeds Food Preference Questionnaire, grip force task, and emotional attentional blink task, administered in a counterbalanced order within each session.

During the test meal, snack food intake task, and the computer tasks, participants were alone in the laboratory, and the experimenter was only present between each part of the procedure to give instructions. Figure 1 illustrates the procedure in each experimental condition. Each participant was debriefed after the end of her second laboratory session.

#### Test Meal

In the fed session, participants were offered a breakfast meal consisting of a porridge and a fruit. Each participant could choose between a couscous (269 kcal) and buckwheat (292 kcal) porridge, and between a banana (∼91 kcal) and an apple [∼88 kcal; the average caloric content of the fruits is based on USDA Food Composition Databases (USDA, 2018)]. Participants were provided with a jug of water and an empty glass. Fifteen minutes were allowed for eating.

#### Snack Food Calorie Intake Task

In the fasted session only, patricipants were offered four kinds of snack foods, 50 g of each: vanilla waffles (552 kcal/100 g), salted peanuts (620 kcal/100 g), sugar-coated raisins (362 kcal/100 g), and pretzels (397 kcal/100 g). Again, a jug of water and an empty glass were provided and participants were given 15 min to eat.

### Hypotheses

We hypothesized that the three tasks show higher food reward scores in the fasted than the fed condition. Further, we expected that the three tasks correlate with each other, with trait binge eating scores, and snack food calorie intake. The hypotheses were preregistered at Open Science Framework^1^. While our preregistered hypothesis also concerned comparing the responsiveness of wanting and liking, as well as the distinction of reactivity to different food categories, these hypotheses were not pursued to preserve the focus of the paper. Note that our current sample size was smaller than planned due to constraints in resources. At the same time, our initial sample size estimate did not account for increases of statistical power for between-subject comparisons gained by having repeated measurements of tasks within each individual, which we now exploit by using repeated measures correlations (Bakdash and Marusich, 2017).

### Statistical Analyses

All analyses were conducted using R software version 3.3.2 (R Core Team, 2013) in RStudio (RStudio Team, 2015). To assess the tasks’ sensitivity to hunger, Student’s *t*-tests were carried out for between-condition comparisons of the LFPQ’s subscales, grip force, and EAB. Additionally, to explore the effect of hunger on food reward scores by food category, linear mixed-effects analyses were performed on the scores of the three tasks. The tests were carried out using the function “lme()” from the package “nlme” (R Core Team et al., 2016). Experimental condition (fasted, fed), order of conditions (fasted first, fed first), food category (high-fat savory, high-fat sweet, low-fat savory, and low-fat sweet), and number of days between the two laboratory sessions were used as independent variables. *Post hoc* comparisons were conducted where significant interaction effects appeared between category and condition, using the Holm–Bonferroni method with the function “lsmeans()” from the package “lsmeans” (Lenth, 2018).

To assess the overall associations between the LFPQ, grip force and EAB tasks across the two measurement sessions, repeated measures correlations were calculated using the function “rmcorr()” from the package “rmcorr” (Bakdash and Marusich, 2017). Repeated measures correlations are better suited than regular correlation analyses for non-independent observations as they make use of repeated measurements within persons, without violating the assumption of independence of observations. Across different food categories, mean scores were calculated for each task by averaging the results of the four food categories.

Additionally, Spearman rank-order correlations were calculated on the task mean scores to explore the correlations between the tasks in fasted and fed condition separately, as well as their correlations with binge eating, BMI, and snack food calorie intake. Between-condition correlations were calculated for each task to describe the strength of the relations between the scores in fasted and fed conditions. A non-parametric correlation coefficient was chosen due to violations of normality.

All reported *p*-values for correlations suggested by hypotheses are one-tailed. Specifically, correlations between the computerized tasks (excluding neutral categories) and their correlations with trait binge eating and snack food intake are one-tailed. All other tests of correlations are two-tailed.

The difference of correlations between two tasks in the fasted and fed condition was calculated by subtracting one correlation from another. *P*-values were empirically estimated with a permutation test. Namely, within both fasted and fed condition, one vector was randomized and then correlated with the non-randomized vector, and difference in correlations between two conditions was found. Repeating this 10,000 times provided a null distribution to compare our empirical results to. Similarly to the correlations described above, this analysis was carried out on mean scores of tasks across four types of food pictures.

## Results

### Participant Characteristics

Out of the 40 women who participated, one reported greater average hunger during the fed than the fasted condition and was excluded from all analyses. The remaining sample of 39 women had a mean age of 25.51 years (*SD* = 5.99, range 18–38 years) and a mean BMI of 22.51 kg/m^2^ (*SD* = 3.58, range 17.59–33.46). Sixteen of the participants had a university degree, one had a secondary school diploma and 22 were in the process of completing their higher education. The average trait binge eating score on EDAS was 12.82 (*SD* = 5.86). Participants consumed an average of 84.42 kcal from salted peanuts (*SD* = 89.23), 104.34 kcal from vanilla waffles (*SD* = 88.6), 53.44 kcal from pretzels (*SD* = 48.91) and 29.47 kcal from sugar-coated raisins (*SD* = 35.87). Mean time between two sessions was 7.92 days (*SD* = 2.84; range 3–18). Four women’s results were excluded from analyses of the grip force task due to a technical error during data recording.

### Hunger Manipulation

**FIGURE 2:**
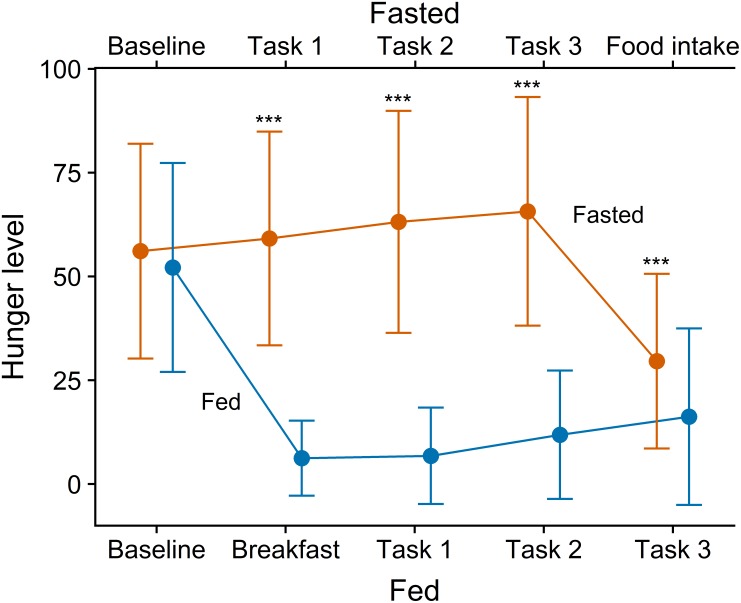
Means and standard errors of subjective hunger ratings during fasted and fed condition. ^∗∗∗^*p* < 0.001 (Wilcoxon signed rank test). Tasks 1, 2, and 3 were the Leeds Food Preference Questionnaire, grip force task, and emotional attentional blink task, administered in a counterbalanced order within each session.

Analysis of subjective hunger VAS ratings confirmed the effectiveness of the hunger manipulation: upon arrival to the lab, no baseline differences were found between the conditions, but hunger was significantly lower following test meal consumption in the fed condition than in the fasted condition (Figure 2).

### Hunger Effects

**FIGURE 3:**
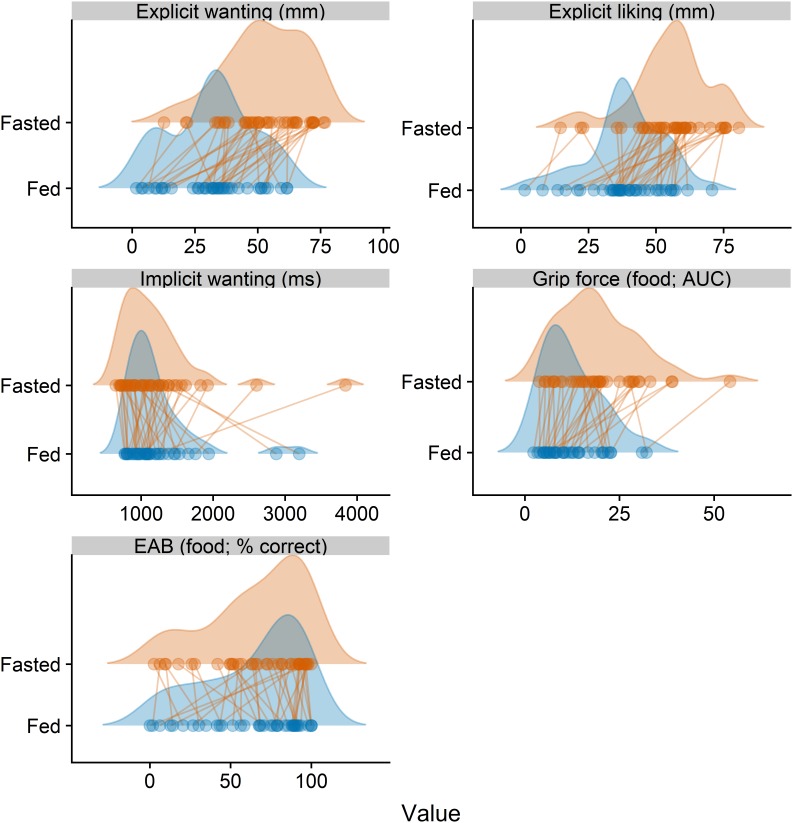
Distributions of averaged scores in the fasted and fed condition. Points represent individual participants and connecting lines the change in score values between two conditions. EAB, emotional attentional blink; AUC, area under the curve.

Student’s *t*-tests conducted to compare the tasks in the fasted and fed state revealed significant differences between the conditions for explicit wanting, explicit liking, and grip force (food category only), suggesting that the scores were significantly higher in the fasted condition. Mean scores for each task, averaged across food categories, are summarized in Table 2 with *t*-statistics and associated significance levels. Figure 3 illustrates the distribution of scores for all subscales of the LFPQ as well as the grip force and EAB tasks for each experimental condition.

**Table 2:** Descriptive statistics and comparisons of the Leeds Food Preference Questionnaire subscales, emotional attentional blink, and grip force task by condition.

Variable	Fasted	Fed	*t*	*p*	*df*
Explicit wanting (mm)	52.70 (16.08)	31.69 (16.84)	7.14	<0.001	38
Explicit liking (mm)	54.99 (14.82)	38.89 (14.56)	6.26	<0.001	38
Implicit wanting (ms)	1208 (587)	1211 (516)	-0.03	0.979	38
EAB (food; % correct)	66 (0.3)	64 (0.31)	0.55	0.583	38
EAB (neutral; % correct)	64 (0.32)	63 (0.31)	0.28	0.783	37
Grip force (food; AUC)	19.42 (10.99)	12.33 (7.62)	4.52	<0.001	34
Grip force (neutral; AUC)	8.16 (7.72)	7.24 (6.72)	1.26	0.215	34


*Means are presented with standard deviations in parentheses. EAB, emotional attentional blink; AUC, area under the curve.*

The linear mixed-effects models enabled us to compare reactivity to different food categories, as well as take into account possible order effects and effects of the time between the two sessions. Main effects of condition (fasted vs. fed) were found for explicit wanting (*F* = 123.31, *p* < 0.001), explicit liking (*F* = 71.13, *p* < 0.001), and grip force (*F* = 44.94, *p* < 0.001) alike, confirming the hunger sensitivity of these measures. Main effects of category were also present: *F* = 12.63, *p* < 0.001 for explicit wanting, *F* = 11.01, *p* < 0.001 for explicit liking, and *F* = 24.01, *p* < 0.001 for grip force, implying differences in scores between different food categories. Statistically significant interactions between food category and condition for explicit wanting (*F* = 10.35, *p* < 0.001), explicit liking (*F* = 9.37, *p* < 0.001), and grip force (*F* = 4.00, *p* = 0.004) revealed that scores changed differently for food categories between conditions. The fact that a difference of grip force was found between the conditions for two of the food categories but not for the neutral category suggests that the hunger manipulation caused a food-specific increase in motivation rather than a heightened reactivity to images in general.

For the reaction times of the LFPQ’s implicit wanting subscale, a significant main effect appeared for category (*F* = 6.58, *p* < 0.001). Neither the condition × category interaction nor the other main effects were significant. This suggests that although the reaction times for choosing between two alternative foods was different between food categories, this forced choice procedure reflected no hunger-dependent change in overall speed of choice.

For the EAB task, no significant main effects were detected. The condition × category interaction was similarly non-significant. These findings suggest that the task was not sensitive to changes of hunger and did not differentiate between image categories. It should be noted that the percentage of correct answers did not differ between any of the image categories, including the neutral category.

Importantly, the main effects of order (fasted first vs. fed first) and time between the sessions were below the level of significance in each of the five models, suggesting that these variables did not meaningfully affect the results. Supplementary Table S1 summarizes all main effects and interactions of the mixed-effect models. Supplementary Figure S1 shows mean scores by image category and condition with *post hoc* Holm–Bonferroni analyses.

### Correlations Between Food Reward Tasks, BMI, Trait Binge Eating, and Snack Food Calorie Intake

Repeated measures correlation analyses showed moderate to strong positive associations between mean scores of explicit wanting, explicit liking, and grip force. Neither EAB scores nor forced choice reaction times had any of the expected correlations with the other tasks, trait binge eating, or snack food calorie intake. Spearman correlations confirmed that the total amount of calories consumed in the snacking task correlated with explicit liking and explicit wanting. The correlations of trait binge eating with explicit wanting, explicit liking, grip force, forced choice reaction times, and EAB scores were non-significant. Correlations between the three tasks, trait binge eating, BMI and snack food intake are summarized in Table 3.

**Table 3:** Correlations between explicit liking, explicit wanting, and implicit wanting (Leeds Food Preference Questionnaire), grip force task, attentional blink task, BMI, trait binge eating, and snack food intake.

Variable	Explicit liking (mm)	Explicit wanting (mm)	Grip force (food; AUC)	Grip force (neutral; AUC)	Implicit wanting (ms)	EAB (food; % correct)	EAB (neutral; % correct)	BMI	Trait binge eating	Calories consumed
Explicit liking (mm)	1	0.92***	0.49**	0.07	-**0.12**	**0.20**	0.14	-0.34*	**0.12**	0.39**
Explicit wanting (mm)		1	0.50***	0.11	-**0.17**	**0.14**	0.11	-0.27	**0.22**	0.45**
Grip force (food; AUC)			1	0.39*	-**0.05**	**0.11**	0.16	-0.07	**0.17**	**0.16**
Grip force (neutral; AUC)				1	0.31	0.08	0.09	0.01	-0.03	-0.17
Implicit wanting (ms)					1	**0.14**	0.03	0.27	**0.05**	-**0.24**
EAB (food; % correct)						1	0.75***	-0.25	**0.06**	**0.38**
EAB (neutral; % correct)							1	-0.12	-0.06	0.24
BMI								1	0.03	-0.31
Binge eating									1	0.40*
Calories consumed										1


*For correlations between computerized tasks, repeated measures correlations are presented; for correlations including BMI, trait binge eating, and snack food calorie intake, Spearman rank-order correlations are shown, with repeated measures of tasks averaged across conditions. One-tailed tests suggested by hypotheses are marked with bold. Range of degrees of freedom is 33–38. EAB, emotional attentional blink; AUC, area under the curve. ^∗∗∗^p < 0.001, ^∗∗^p < 0.01, ^∗^p < 0.05.*

Table 4 shows between-condition correlations for each task, as well as correlations between the tasks in each condition. Between-condition correlations were moderate to strong for the LFPQ subscales, as well as grip force and EAB. Supplementary Table S2 shows a breakdown of correlations by food category.

**Table 4:** Between-condition correlations and correlations between the tasks.

Variable	Explicit liking (mm)	Explicit wanting (mm)	Grip force (food; AUC)	Grip force (neutral; AUC)	Implicit wanting (ms)	EAB (food; % correct)	EAB (neutral; % correct)
Explicit liking (mm)	0.36*	0.82***	0.07	-0.29	-0.09	0.06	0.02
Explicit wanting (mm)	0.9***	0.36*	0.10	-0.29	-0.10	0.12	0.07
Grip force (food; AUC)	0.55***	0.54**	0.61***	0.71***	-0.26	-0.03	-0.10
Grip force (neutral; AUC)	0.17	0.19	0.39*	0.69***	-0.27	-0.19	-0.30
Implicit wanting (ms)	-0.20	-0.08	0.01	0.03	0.50**	0.09	0.16
EAB (food; % correct)	-0.10	-0.08	-0.15	-0.09	-0.15	0.57***	0.83***
EAB (neutral; % correct)	-0.07	0.00	-0.12	-0.04	-0.16	0.92***	0.43**


*Numbers above the diagonal show correlations in the fasted condition, below the diagonal are correlations for the fed condition. Between-condition correlations for each measure are shown on the diagonal. All correlations are Spearman’s rhos. Range of degrees of freedom is 34–38. EAB, emotional attentional blink; AUC, area under the curve. ^∗∗∗^p < 0.001, ^∗∗^p < 0.01, ^∗^p < 0.05.*

### Between-Condition Comparison of Correlations

Comparisons of correlations between fasted and fed conditions revealed differences of correlations for explicit wanting and grip force (*p* < 0.01) and explicit liking and grip force (*p* < 0.01) with the associations being stronger in the fed condition. Because neither EAB results nor implicit wanting reaction times correlated with the other tasks, comparative procedures were not carried out on the scores of these scales.

## Discussion

While many food reward tasks have been developed, little is known about the extent of their overlap and their associations with other eating-related variables. The current study tested the empirical similarity of three food reward tasks, as well as their responsivity to a hunger manipulation. Namely, we compared the Leeds Food Preference Questionnaire (LFPQ) – a procedure comprised of explicit wanting and liking scales and a forced choice implicit wanting task; a hand grip force task, and an emotional attentional blink (EAB) task. Healthy female participants completed the three tasks after fasting and in a fed state. We found that the tasks higher in explicitness (the LFPQ’s explicit subscales and the grip force task) were responsive to hunger, whereas the more implicit tasks (EAB and implicit wanting reaction times) were not. The explicit tasks also correlated positively with each other, and this effect was amplified in the fed condition. Meanwhile, the implicit tasks did not correlate with the other tasks. These important similarities between explicit wanting, liking, and grip force suggest a significant overlap of the explicit tasks. Finally, subjective reports of explicit wanting and liking also correlated with energy intake in a laboratory-based snack food intake task.

### Hunger Sensitivity

As we anticipated, explicit wanting, liking, and grip force were higher in the fasted than fed condition. This hunger effect was likely food-specific rather than general, as grip force scores only differed between the conditions for food categories, but not for the neutral image category. Contrary to expectations, neither the ability to correctly identify target stimuli in the EAB task nor reaction times in the forced choice procedure significantly differed between the conditions. The lack of a state-dependent change in reaction times contrasts with the previous finding that the speed of choosing between two foods was faster after participants consumed a test meal (Finlayson et al., 2008). However, order effects may have affected the results found by Finlayson and colleagues; this was avoided in the current study as the order of experimental conditions was counterbalanced. Still, because the forced choice task was able to distinguish between food categories that differ in taste and fat content, this task is likely suitable for capturing relative preference. Because the forced choice task is less likely influenced by the cognitive interpretation of perceived wanting (Finlayson et al., 2009), it may be a good addition to more explicit tasks. Together, these results partially confirm our first hypothesis, supporting the idea that the explicit subscales of the LFPQ and the grip force task can capture changes in the motivational component of food reward.

While the EAB should capture automatic attentional processes in eating behavior, we were unable to demonstrate the task’s responsiveness to hunger in the current setup. Presenting stimuli on white backgrounds may have made them easily detectable, obscuring the effect. Alternatively, it is plausible that an attentional bias for food is only present in some subpopulations. For example, it has been shown that gaze duration for food images is increased in obese and normal-weight individuals alike when fasted, but when fed, the increased attention to food is only sustained in persons with obesity (Castellanos et al., 2009). As our sample consisted of healthy women with varying BMIs, effects characteristic to only certain groups of people may have gone undetected. Furthermore, attentional processes to rewarding stimuli may be biased by several traits (e.g., impulsivity) or conditions (e.g., drug dependency, depression; Anderson, 2016). Because many factors contribute to reward-driven attentional processes, very strict control over confounds may be necessary to find a true effect.

### Correlations Between Tasks

Explicit wanting, explicit liking, and grip force were moderately positively correlated with each other in repeated measures analyses, revealing a significant overlap between the tasks. EAB scores and implicit wanting reaction times did not correlate with any of the other tasks used. To conclude, the data partially supported our second hypothesis. The results also indicate that data obtained with the LFPQ and grip force task can likely be pooled, as has been done when comparing different eating-related traits (Vainik et al., 2019) or explicit food reward tasks (Oustric et al., 2018). Comparisons of correlations revealed that the associations between the LFPQ explicit subscales and grip force were stronger in the fed condition, suggesting that individual differences in food reward may best be captured in non-hungry individuals. This is consistent with the view that individual differences should be tested in a non-deprived state because hunger tends to affect everybody in a similar way (Epstein et al., 2007): when hungry, people naturally tend to seek out and consume food, but individual differences in the tendency to eat in a non-hungry state may vary to a great extent.

### Correlations With Snack Food Calorie Intake

Snack food intake correlated positively with explicit wanting and liking measured with the LFPQ, but the expected correlations with grip force, implicit wanting reaction times, and the EAB task were absent, providing partial support for our hypothesis about the tasks’ correlations with calorie consumption. Given that grip force correlates with explicit liking and wanting and is reactive to hunger, the absence of a correlation between grip force and snack food calorie intake may appear surprising. However, a similar result emerged in a previous study where desire to eat (a self-report measure) was superior to a bar-pressing task (a work-for-food task) in predicting *ad libitum* food intake (Rogers and Hardman, 2015). These findings highlight a possible difference between tasks that rely on self-report versus physical effort: while subjective report may be more suitable for predicting intake in a setting where food is readily available (as was in the current study), work-for-food tasks may be specific to the amount of effort one might expend to obtain the reward, rather than the amount of food consumed. This idea, however, remains to be tested.

### Correlations With Trait Binge Eating

Contrary to our hypothesis, none of the tasks used in the current study significantly correlated with trait binge eating. This is inconsistent with reports that trait binge eating is associated with a higher level of wanting and liking for food (Finlayson et al., 2011). Although food reward likely contributes to binge eating, its effect on the tendency to binge eat may appear in combination with a deficit in self-regulatory control processes that limit calorie intake (Berner et al., 2017). If a combination of heightened reward and a deficiency in control processes is a necessary precursor for binge eating, the absence of a correlation should not necessarily be interpreted as a non-existent link between food reward and binge eating. Further research is needed to clarify the nature of the association between these variables.

### Limitations and Future Directions

An important limitation of the current study is low power for between-participant analyses. For between-task correlation analyses, this was remedied by pooling task scores of the fasted and fed condition. However, this was not an option for analyses involving snack food intake, BMI and trait binge eating. As such, these results should be interpreted with caution: moderate to low correlations could have gone undetected. Therefore, we encourage similar research with bigger samples that also measure covariates which may moderate the associations or enable subgroup comparisons. Future studies aiming to clarify the links between food reward tasks might also include other types of tasks not tested in the current study. We believe that the current analysis was a necessary first step in assessing the overlap between food reward tasks and current results can be incorporated into future meta-analyses on the topic.

All in all, the current study provides a comparison of different tasks used in food reward research. Although the three tasks used constitute only a sample of all suggested tasks, they nevertheless represent a diverse selection of approaches to food reward measurement. We hope that our results inspire future studies and aid in the integration of existing literature.

## Ethics Statement

This study was carried out in accordance with the recommendations of Research Ethics Committee of the University of Tartu with written informed consent from all subjects. All subjects gave written informed consent in accordance with the Declaration of Helsinki. The protocol was approved by the Research Ethics Committee of the University of Tartu (application 254/T-12).

## Author Contributions

KA, KK, and UV contributed to conception and design of the study. KA collected data and organized the database, performed the statistical analysis, and wrote the first draft of the manuscript. UV wrote sections of the manuscript. All authors contributed to data analysis and discussion of results, manuscript revision, read, and approved the submitted version.

## Conflict of Interest Statement

The authors declare that the research was conducted in the absence of any commercial or financial relationships that could be construed as a potential conflict of interest. Part of the food offered to participants was donated by AS Kalev. The company had no role in study conception, execution, or reporting.

## Footnotes

1 https://osf.io/afppu/register/5771ca429ad5a1020de2872e

## Supplementary Material

Data collected is available at https://osf.io/njqgy/.

## References

[B1] Akkermann K. Herik M. Aluoja A. Järv A. (2010). *Constructing an Assessment Scale for Eating Disorders.* Tartu: Department of Psychology of University of Tartu.

[B2] Alonso-Alonso M. Woods S. C. Pelchat M. Grigson P. S. Stice E. Farooqi S. (2015). Food reward system: current perspectives and future research needs. *Nutr. Rev.* 73 296–307. 10.1093/nutrit/nuv002 26011903 PMC4477694

[B3] Anderson B. A. (2016). The attention habit: how reward learning shapes attentional selection. *Ann. N.Y. Acad. Sci.* 1369 24–39. 10.1111/nyas.12957 26595376

[B4] Bakdash J. Z. Marusich L. R. (2017). Repeated measures correlation. *Front. Psychol.* 8:456. 10.3389/fpsyg.2017.00456 28439244 PMC5383908

[B5] Berner L. A. Winter S. R. Matheson B. E. Benson L. Lowe M. R. (2017). Behind binge eating: a review of food-specific adaptations of neurocognitive and neuroimaging tasks. *Physiol. Behav.* 176 59–70. 10.1016/j.physbeh.2017.03.037 28363840 PMC5695923

[B6] Castellanos E. H. Charboneau E. Dietrich M. S. Park S. Bradley B. P. Mogg K. (2009). Obese adults have visual attention bias for food cue images: evidence for altered reward system function. *Int. J. Obes.* 33 1063–1073. 10.1038/ijo.2009.138 19621020

[B7] Dale G. Arnell K. (2013). How reliable is the attentional blink? Examining the relationships within and between attentional blink tasks over time. *Psychol. Res.* 77 99–105. 10.1007/s00426-011-0403-y 22159732

[B8] Dalton M. Finlayson G. (2014). Psychobiological examination of liking and wanting for fat and sweet taste in trait binge eating females. *Physiol. Behav.* 136 128–134. 10.1016/j.physbeh.2014.03.019 24662699

[B9] Dux P. E. Marois R. (2009). The attentional blink: a review of data and theory. *Atten. Percept. Psychophys.* 71 1683–1700. 10.3758/APP.71.8.1683 19933555 PMC2915904

[B10] Emery R. L. Levine M. D. (2017). Questionnaire and behavioral task measures of impulsivity are differentially associated with body mass index: a comprehensive meta-analysis. *Psychol. Bull.* 143 868–902. 10.1037/bul0000105 28493725 PMC13051558

[B11] Epstein L. H. Leddy J. J. Temple J. L. Faith M. S. (2007). Food reinforcement and eating: a multilevel analysis. *Psychol. Bull.* 133 884–906. 10.1037/0033-2909.133.5.884 17723034 PMC2219695

[B12] Field M. Werthmann J. Franken I. Hofmann W. Hogarth L. Roefs A. (2016). The role of attentional bias in obesity and addiction. *Health Psychol.* 35 767–780. 10.1037/hea0000405 27505196

[B13] Finlayson G. Arlotti A. Dalton M. King N. Blundell J. E. (2011). Implicit wanting and explicit liking are markers for trait binge eating. A Susceptible Phenotype for Overeating. *Appetite* 57 722–728. 10.1016/j.appet.2011.08.012 21896296

[B14] Finlayson G. Bryant E. Blundell J. E. King N. A. (2009). Acute compensatory eating following exercise is associated with implicit hedonic wanting for food. *Physiol. Behav.* 97 62–67. 10.1016/j.physbeh.2009.02.002 19419671

[B15] Finlayson G. King N. Blundell J. (2008). The role of implicit wanting in relation to explicit liking and wanting for food: implications for appetite control. *Appetite* 50 120–127. 10.1016/j.appet.2007.06.007 17655972

[B16] Finlayson G. King N. Blundell J. E. (2007). Is it possible to dissociate ‘liking’ and ‘wanting’ for foods in humans? A novel experimental procedure. *Physiol. Behav.* 90 36–42. 10.1016/j.physbeh.2006.08.020 17052736

[B17] French S. A. Mitchell N. R. Finlayson G. Blundell J. E. Jeffery R. W. (2014). Questionnaire and laboratory measures of eating behavior. Associations with energy intake and BMI in a community sample of working adults. *Appetite* 72 50–58. 10.1016/j.appet.2013.09.020 24096082 PMC3893825

[B18] Griffioen-Roose S. Mars M. Finlayson G. Blundell J. E. de Graaf C. (2011). The effect of within-meal protein content and taste on subsequent food choice and satiety. *Br. J. Nutr.* 106 779–788. 10.1017/S0007114511001012 21736849

[B19] Herik M. (2009). *Söömishäirete Hindamise Skaala Konstrueerimine*. Available at: http://rahvatervis.ut.ee/handle/1/2163 (accessed December 14, 2018).

[B20] Higgs S. Spetter M. S. (2018). Cognitive control of eating: the role of memory in appetite and weight gain. *Curr. Obes. Rep.* 7 50–59. 10.1007/s13679-018-0296-9 29430616 PMC5829122

[B21] Kelly A. J. Dux P. E. (2011). Different attentional blink tasks reflect distinct information processing limitations: an individual differences approach. *J. Exp. Psychol. Hum. Percept. Perform.* 37 1867–1873. 10.1037/a0025975 22004195

[B22] Kelley T. L. (1927). *Interpretation of Educational Measurements.* Oxford: World Book Co.

[B23] Lang P. J. (2005). International Affective Picture System (IAPS): Affective Ratings of Pictures and Instruction Manual. Technical Report A-8 Gainesville, FL: University of Florida.

[B24] Lenth R. (2018). *lsmeans: Least-Squares Means.* Available at: https://CRAN.R-project.org/package=lsmeans (accessed December 14, 2018).

[B25] Mason A. E. Vainik U. Acree M. Tomiyama A. J. Dagher A. Epel E. S. (2017). Improving assessment of the spectrum of reward-related eating: the RED-13. *Front. Psychol.* 8:795. 10.3389/fpsyg.2017.00795 28611698 PMC5447741

[B26] MATLAB (2016). *MATLAB.* Natick, MA: The MathWorks Inc.

[B27] McHugo M. Olatunji B. O. Zald D. H. (2013). The emotional attentional blink: what we know so far. *Front. Hum. Neurosci* 7:151. 10.3389/fnhum.2013.00151 23630482 PMC3632779

[B28] Michaud A. Vainik U. García-García I. Dagher A. (2017). Overlapping neural endophenotypes in addiction and obesity. *Front. Endocrinol.* 8:127. 10.3389/fendo.2017.00127 28659866 PMC5469912

[B29] Nijs I. M. T. Franken I. H. A. (2012). Attentional processing of food cues in overweight and obese individuals. *Curr. Obes. Rep.* 1 106–113. 10.1007/s13679-012-0011-1 22611523 PMC3342487

[B30] Oustric P. Gibbons C. Beaulieu K. Blundell J. Finlayson G. (2018). Changes in food reward during weight management interventions – a systematic review. *Obes. Rev.* 19 1642–1658. 10.1111/obr.12754 30144275

[B31] Piech R. M. Pastorino M. T. Zald D. H. (2010). All I saw was the cake. Hunger effects on attentional capture by visual food cues. *Appetite* 54 579–582. 10.1016/j.appet.2009.11.003 19914320

[B32] Price M. Higgs S. Lee M. (2015). Self-reported eating traits: underlying components of food responsivity and dietary restriction are positively related to BMI. *Appetite* 95 203–210. 10.1016/j.appet.2015.07.006 26162952

[B33] Psychology Software Tools Inc (2012). *E-Prime 2.0.* Sharpsburg: Psychology Software Tools, Inc.

[B34] R Core Team (2013). *R: A Language and Environment for Statistical Computing.* Vienna: R Foundation for Statistical Computing.

[B35] R Core Team Pinheiro J. Bates D. DebRoy S. Sarkar D. (2016). *nlme: linear and nonlinear mixed effects models.* Vienna: R Core Team.

[B36] Raymond J. E. Shapiro K. L. Arnell K. M. (1992). Temporary suppression of visual processing in an RSVP task: an attentional blink? *J. Exp. Psychol. Hum. Percept. Perform.* 18 849–860. 10.1037/0096-1523.18.3.849 1500880

[B37] Roberts H. C. Denison H. J. Martin H. J. Patel H. P. Syddall H. Cooper C. (2011). A review of the measurement of grip strength in clinical and epidemiological studies: towards a standardised approach. *Age Ageing* 40 423–429. 10.1093/ageing/afr051 21624928

[B38] Rogers P. J. Hardman C. A. (2015). Food reward. What it is and how to measure it. *Appetite* 90 1–15. 10.1016/j.appet.2015.02.032 25728883

[B39] Rolls E. T. (2015). Taste, olfactory, and food reward value processing in the brain. *Prog. Neurobiol.* 127–128, 64–90. 10.1016/j.pneurobio.2015.03.002 25812933

[B40] RStudio Team (2015). *RStudio: Integrated Development for R.* Boston, MA: RStudio, Inc.

[B41] Stevenson R. J. (2017). Psychological correlates of habitual diet in healthy adults. *Psychol. Bull.* 143 53–90. 10.1037/bul0000065 27618545

[B42] Sultson H. Kukk K. Akkermann K. (2017). Positive and negative emotional eating have different associations with overeating and binge eating: construction and validation of the positive-negative emotional eating scale. *Appetite* 116 423–430. 10.1016/j.appet.2017.05.035 28549761

[B43] Temple J. L. (2014). Factors that influence the reinforcing value of foods and beverages. *Physiol. Behav.* 136 97–103. 10.1016/j.physbeh.2014.04.037 24793218 PMC5904513

[B44] Uher R. Treasure J. Heining M. Brammer M. J. Campbell I. C. (2006). Cerebral processing of food-related stimuli: effects of fasting and gender. *Behav. Brain Res.* 169 111–119. 10.1016/j.bbr.2005.12.008 16445991

[B45] USDA (2018). *Food Composition Databases Show Foods List* Available at: https://ndb.nal.usda.gov/ndb/search/list (accessed December 14, 2018).

[B46] Vainik U. Dagher A. Dubé L. Fellows L. K. (2013). Neurobehavioural correlates of body mass index and eating behaviours in adults: a systematic review. *Neurosci. Biobehav. Rev.* 37 279–299. 10.1016/j.neubiorev.2012.11.008 23261403 PMC4017079

[B47] Vainik U. García-García I. Dagher A. (2019). Uncontrolled eating: a unifying heritable trait linked with obesity, overeating, personality and the brain. *Eur. J. Neurosci.* 9 1–16. 10.1111/ejn.14352 30667547

[B48] Vainik U. Meule A. (2018). Jangle fallacy epidemic in obesity research: a comment on Ruddock et al. (2017). *Int. J. Obes.* 42 585–586. 10.1038/ijo.2017.264 29081502

[B49] Vainik U. Neseliler S. Konstabel K. Fellows L. K. Dagher A. (2015). Eating traits questionnaires as a continuum of a single concept. Uncontrolled eating. *Appetite* 90 229–239. 10.1016/j.appet.2015.03.004 25769975

[B50] Wang G.-J. Volkow N. D. Telang F. Jayne M. Ma Y. Pradhan K. (2009). Evidence of gender differences in the ability to inhibit brain activation elicited by food stimulation. *Proc. Natl. Acad. Sci. U.S.A.* 106 1249–1254. 10.1073/pnas.0807423106 19164587 PMC2633545

[B51] Werthmann J. Jansen A. Roefs A. (2015). Worry or craving? A selective review of evidence for food-related attention biases in obese individuals, eating-disorder patients, restrained eaters and healthy samples. *Proc. Nutr. Soc.* 74 99–114. 10.1017/S0029665114001451 25311212

[B52] Yang Y. Shields G. S. Guo C. Liu Y. (2018). Executive function performance in obesity and overweight individuals: a meta-analysis and review. *Neurosci. Biobehav. Rev.* 84 225–244. 10.1016/j.neubiorev.2017.11.020 29203421

[B53] Ziauddeen H. Subramaniam N. Cambridge V. C. Medic N. Farooqi I. S. Fletcher P. C. (2014). Studying food reward and motivation in humans. *J. Vis. Exp.* 85:e51281. 10.3791/51281 24686284 PMC4153444

[B54] Ziauddeen H. Subramaniam N. Gaillard R. Burke L. K. Farooqi I. S. Fletcher P. C. (2012). Food images engage subliminal motivation to seek food. *Int. J. Obes.* 36 1245–1247. 10.1038/ijo.2011.239 22143617 PMC3438467

