# Supplementary material for: Assessing the Overlap Between Three Measures of Food Reward
Source: Front Psychol. 2019 Apr 26;10:883. doi: 10.3389/fpsyg.2019.00883 (PMC6524717; doi:10.3389/fpsyg.2019.00883)
Supplement: Supplementary file 3 [file Image_1.pdf]

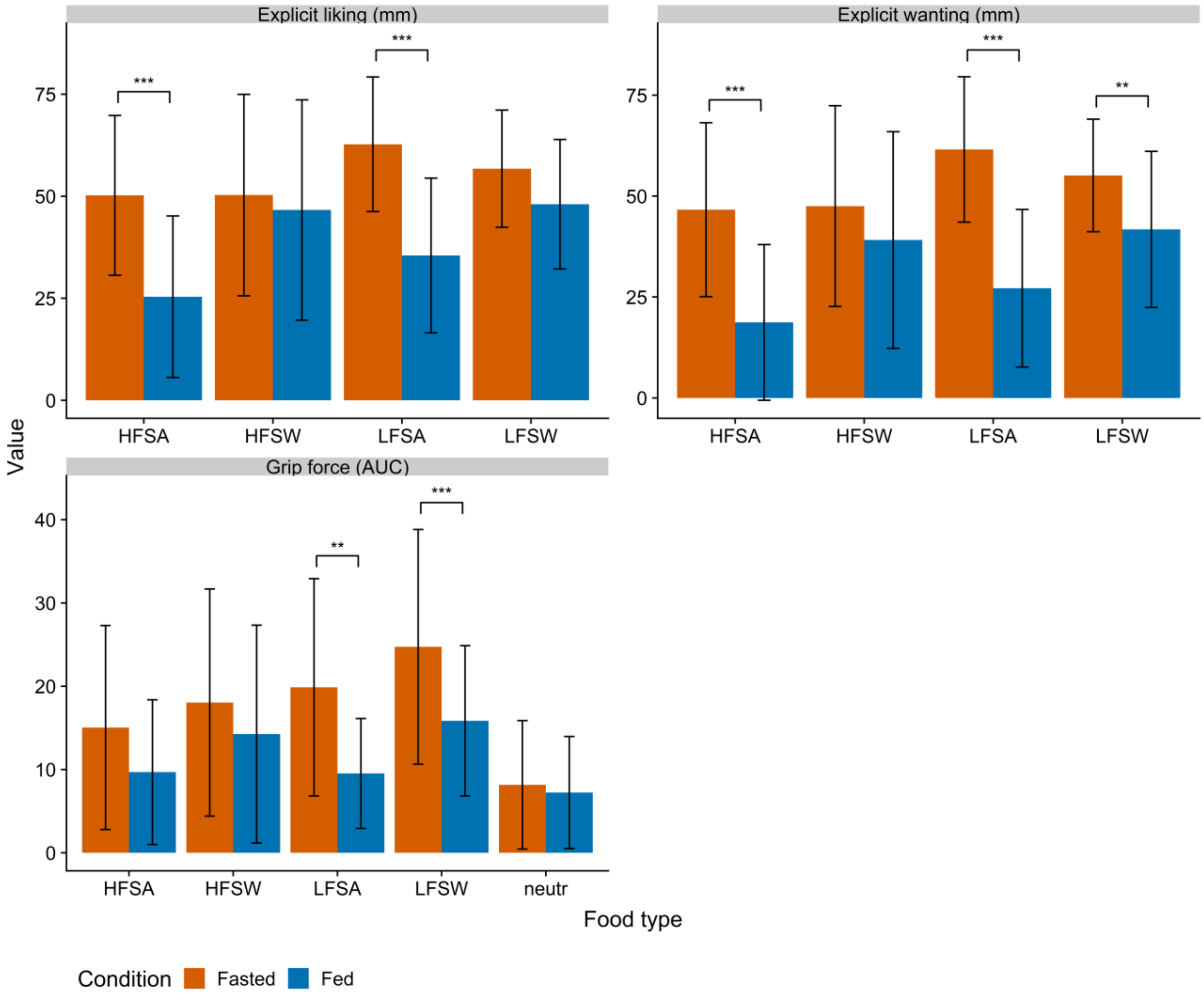

FIGURE 1S | Means and standard deviations of task score values by condition and food category. The differences indicated are based on Holm-Bonferroni comparisons. AUC, area under the curve; HFSA, high-fat savoury; HFSW, high-fat sweet; LFSA, low-fat savoury; LFSW, low-fat sweet. *** $p < 0.001$, ** $p < 0.01$.

Arumäe, K., Kreegipuu, K., & Vainik, U. (2019). Assessing the overlap between three measures of food reward. *Frontiers in Psychology*, *10*. https://doi.org/10.3389/fpsyg.2019.00883
